# Radiological assessment of skeletal muscle index and myosteatosis and their impact postoperative outcomes after liver transplantation

**DOI:** 10.2478/raon-2023-0025

**Published:** 2023-06-21

**Authors:** Miha Petric, Taja Jordan, Popuri Karteek, Sabina Licen, Blaz Trotovsek, Ales Tomazic

**Affiliations:** Department of Abdominal Surgery, University Medical Centre Ljubljana, Ljubljana, Slovenia; Faculty of Medicine, University of Ljubljana, Ljubljana, Slovenia; Institute of Radiology, University Medical Centre Ljubljana, Ljubljana, Slovenia; Department of Computer Science, Memorial University of Newfoundland, St. John's, NL, Canada; Faculty of Health Sciences, University of Primorska, Izola, Slovenia

**Keywords:** muscle mass, liver transplantation, myosteatosis, skeletal muscle index, GLIM score

## Abstract

**Background:**

Liver transplantation offers curative treatment to patients with acute and chronic end-stage liver disease. The impact of nutritional status on postoperative outcomes after liver transplantation remains poorly understood. The present study investigated the predictive value of radiologically assessed skeletal muscle index (SMI) and myosteatosis (MI) on postoperative outcomes.

**Patients and methods:**

Data of 138 adult patients who underwent their first orthotopic liver transplantation were retrospectively analysed. SMI and MI in computer tomography (CT) scan at the third lumbar vertebra level were calculated. Results were analyzed for the length of hospitalisation and postoperative outcomes.

**Results:**

In 63% of male and 28.9% of female recipients, low SMI was found. High MI was found in 45(32.6%) patients. Male patients with high SMI had longer intensive care unit (ICU) stay (P < 0.025). Low SMI had no influence on ICU stay in female patients (P = 0.544), length of hospitalisation (male, P > 0.05; female, P = 0.843), postoperative complication rates (males, P = 0.883; females, P = 0.113), infection rate (males, P = 0.293, females, P = 0.285) and graft rejection (males, P = 0.875; females, P = 0.135). The presence of MI did not influence ICU stay (P = 0.161), hospitalization (P = 0.771), postoperative complication rates (P = 0.467), infection rate (P = 0.173) or graft rejection rate (P = 0.173).

**Conclusions:**

In our study, changes in body composition of liver transplant recipients observed with SMI and MI had no impact on postoperative course after liver transplantation. CT body composition analysis of recipients and uniformly accepted cut-off points are crucial to producing reliable data in the future.

## Introduction

Since 1963 when the first liver transplantation (LT) was performed by Starzl^1^, it has become a standard treatment modality for patients with acute liver failure and chronic liver disease.^2^ Ninety percent 5-year survival rate and better quality of life are the two most important outcomes of LT.^2^ Most liver transplant centres use the Model for End-stage Liver Disease (MELD) score for organ allocation.^3^ Patients with MELD score 15 or more, patients with poor quality of life due to chronic liver disease symptoms (diuretic-intractable ascites, variceal bleeding, pruritus, cachexia), and patients with acute liver failure are those who benefit most from LT.^3^ MELD score can underestimate the severity of liver disease in specific groups of patients (acute on chronic liver disease, presence of sarcopenia, chronic kidney disease, etc.).^3^ Several modifications of the MELD score have been introduced, but none offers a more reliable and accurate scoring system. Albumin-bilirubin (ALBI) score is mainly used as an objective method to assess liver function and predict postoperative complications, particularly after hepatectomy in patients with hepatocellular carcinoma (HCC).^4^ Its role in determining post-LT outcomes is not yet determined. Two main objective parameters of nutritional status are sarcopenia and myosteatosis (MI). The European Working Group on Sarcopenia defines sarcopenia as the presence of low muscle mass (under the 5^th^ percentile) and low muscle function (strength or performance) in patients with advanced age, cancer, or other diseases.^5,6^ Myosteatosis is defined as the abnormal fatty transformation of skeletal muscle. It negatively affects muscle strength and is common in advanced age^7,8^, diabetes^7,8^, obesity^9,10^, chronic^9^, and malignant diseases.^11,12^ Overview of the literature shows large number of different methods used for body composition assessment in patients with liver cirrhosis.^13^ There is still no consensus on the best tools for each body component in patients with liver cirrhosis. Most frequently used are computed tomography (CT), bioimpedance analysis (BIA), dual-energy X-ray absorptiometry (DXA) and anthropometry.^13^ Some of them BIA, DXA and body mass index (BMI)) are not applicable in patients with end-stage liver disease due to frequent water retention.^14,15^ In the last decade, computed tomography with automated or semiautomated body composition analysis at the third lumbar vertebra has emerged as an objective method of defining the nutritional status of patients with chronic liver disease.^15,16^ Nutritional assessment with CT is not affected by water retention or presence of ascites. Skeletal muscle volume and myosteatosis can be measured from CT images obtained as a part of routine pre-transplant evaluation.^14^ Skeletal muscle mass index (SMI) is calculated as muscle mass area divided by the square of the height. The Global Leadership Initiative on Malnutrition (GLIM)^16^ score was introduced as a potential nutritional assessment tool in recent years. It was shown to have good predictive value as a risk assessment tool for postoperative morbidity and mortality in patients after colorectal surgery.^17^ However, its role as a predictive factor in LT is not yet established.

This study aimed to investigate feasibility of radiological assessment of a nutritional status of a patient and the predictive value of SMI and MI on postoperative complications, length of hospitalization, liver graft rejection, and mortality. We further compared the predictive value of SMI and MI with MELD, ALBI, and GLIM scores.

## Patients and methods

We retrospectively analyzed 138 adult patients who had first orthotropic LT from brain dead donors between 1.1.2012 and 1.1.2020 in our institution. We excluded patients who had re-LT procedure and those whose abdominal CT scan could not be obtained from data base or received reduced size and liver graft from donation after cardiac death (DCD) donor. From the medical database, we collected recipient age, gender, body mass index, underlying liver disease, presence of ascites, hepatocellular carcinoma (HCC), and laboratory parameters (serum levels of sodium, creatinine, albumin, protein, bilirubin, and International normalized ratio (INR)). We calculated MELD and ALBI scores from laboratory parameters. Among GLIM criteria, we used chronic liver failure and end-stage liver disease as etiologic and SMI or MI as phenotypic criteria. Length of intensive care unit (ICU) stay, hospitalization, postoperative complications according to Clavien-Dindo classification^18^, infections, and 90-day mortality were collected from the database of liver recipients and analyzed. Diagnosis of liver rejections was confirmed with laboratory tests and histological examination of all liver graft specimens obtained by ultrasound-guided biopsy. Acute rejection was defined with 6 points or more according to the liver allograft fibrosis score.^19^

The Slovenian National Medical Ethics Committee approved our study design (approval number 0120–230/2018–10) and waived the need to obtain informed consent from participants.

### CT-body composition analysis

Abdominal CT scans were obtained from the hospital database system. In case of multiple CT scans, we used last CT scan before LT procedure. A single slice of each patient at the level of the 3^rd^ lumbar vertebrae was selected for automatic segmentation. CT scans were analysed using the “Automated Body Composition Analyzer using Computed tomography image Segmentation” (ABACS)^20^ software, which uses predefined Hounsfield units (HU) values to recognize different tissues. ABACS uses HU values from −29 to +150 HU to assess and calculate the total cross-sectional area for muscular tissue (SMA – skeletal muscle area). The L3 skeletal muscles included the psoas muscle, the lumbar muscles, the erector spinae, the transversus abdominis muscle, the internal and external oblique muscles, and the rectus abdominis. SMI was calculated using the following formula: SMI = SMA (cm^2^) / height2 (m^2^)^9,21^ and patients were divided into a group with lower SMI (men < 52.4 cm^2^/m^2^, women < 38 cm^2^/m^2^)^9,21^ and another with normal SMI. MI was determined by the medium value of HU in a skeletal muscle area. We used recently defined threshold parameters for MI in a patient with a chronic liver disease (< 33 HU in patients with a BMI ≥ 25 kg/m^2^ and < 41 HU in those with a BMI < 25).^22,23,24^ Based on previous literature findings and conclusions no adjustment for sex was made for MI.^23,24^

### Statistical analysis

Data were analysed using SPSS for macOS, 26^th^ edition. Descriptive statistics such as frequencies, percentages, mean/median, and standard deviations were used for description and summary. Because the data were not normally distributed, patient characteristics were compared between groups using the Kruskal-Wallis test and the Mann-Whitney U test. In addition, Spearman rank-order correlation and multiple linear regression were used to determine the relationship between variables and predict patient outcomes based on their characteristics and condition, as well as the chi-square independence test. A P-value ≤ 0.05 was considered statistically significant.

## Results

### Patients characteristics

Between 1.1.2012 and 1.1.2020, 138 patients (100 men and 38 women) met the criteria for inclusion in the study. The median age was 57.5 years (22 to 69 years). Table 1 provides general data on the population laboratory and clinical variables.

**TABLE 1.** Laboratory and clinical data of patients

**Variable**	**Min**	**Max**	**IQR**	**SD**	**95% CI**	

					**Lower**	**Upper**
BMI	15	38	6	4.775	25.13	26.74
Waiting time for liver transplantation (days)	1	691	166	151.311	108.44	159.38
Sodium	121	147	6	4.701	136.09	137.68
Creatinine	41	696	46	67.082	85.91	108.49
Albumin	10	62	10	7.674	31.28	33.86
Protein	23	89	12	11.494	65.80	69.74
Bilirubin	2	687	56	107.535	51.83	88.04
INR	1	4	1	.502	1.44	1.61
MELD score	7	46	9	6.700	14.30	16.56
ALBI score	−4	0	1.13	0.802	−1.87	−1.60

ALBI score = albumin-bilirubin score; BMI = body mass index; INR = international normalized ratio; IQR = Interquartile range; MELD score = model for end-stage liver disease score

Sixty-one (61, 44.2%) patients had LT due to inherited or metabolic liver disease, 60 patients had alcoholic liver disease (43.5%), and 17 patients had virus-related liver disease (12.3%). At the time of LT, 80 (58%) patients had ascites, and 27 patients had HCC (19.4%).

### Incidence of low SMI and MI

In our study group, 63% of male and 28.9% of female patients had low SMI. MI was present in 45 (32.6%) patients. We found no statistical significance between the aetiology of underlying liver disease and SMI (*P* = 0.214). The aetiology of liver disease had a statistically significant influence on the incidence of MI. Patients with alcoholic aetiology had more fatty infiltrated muscles than patients with other liver disease aetiologies (*P* = 0.008) (Table 2).

**TABLE 2.** Aetiology of liver disease and incidence of myosteatosis

**Variable**		**Myosteatosis**	

		**no**	**yes**
Liver failure	Alcohol-related	32	28
	Virus-related	14	3
	Other	47	14
*χ^2^*	9.717		
Degrees of freedom (Df)	2		
*p*	*0.008*		

A Spearman's rank-order correlation was run to determine the relationship between the MI and BMI. There was a moderate, negative correlation between MI and BMI, which was statistically significant (rs = −0.597, p < 0.000).

**FIGURE 1.**
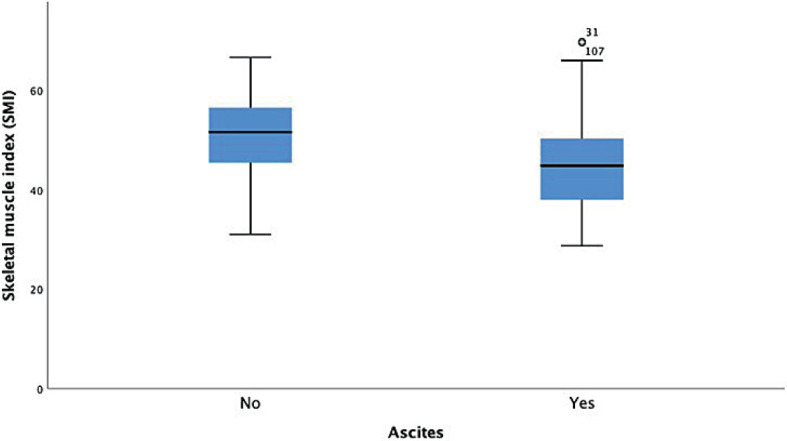
Correlation between ascites and skeletal muscle index.

Eighty patients (57.9%) had decompensated liver cirrhosis with the presence of ascites at the time of CT scan. The Mann-Whitney U test indicates that ascites significantly impacted patients’ SMI. The presence of ascites correlated with low SMI (*P* < 0.05, Figure 1) and had no impact on the incidence of MI (*P* = 0.244).

Presence of HCC in patients with liver disease (27 patients, 19.6%) had no influence on SMI (*P* = 0.546) or MI (*P* = 0.174).

### Influence of low SMI on hospitalization

**FIGURE 2.**
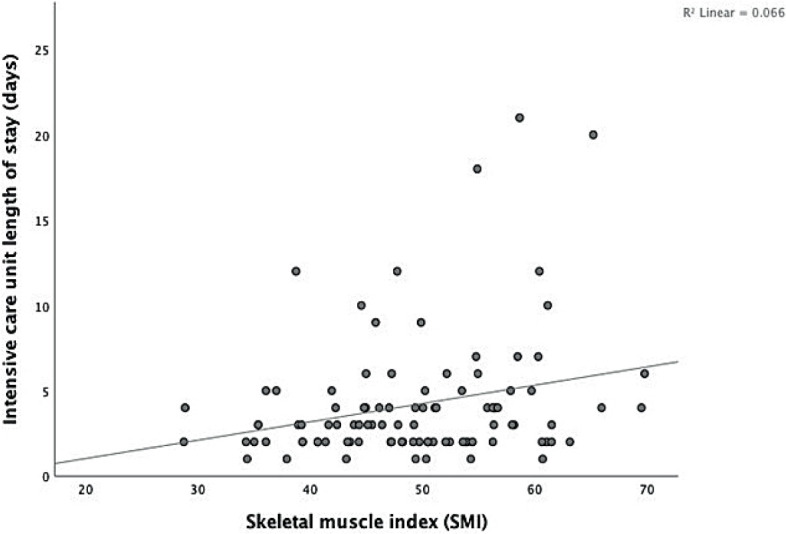
Scatter diagram showing a positive correlation between the intensive care unit length of stay (days) and skeletal muscle index.

The Mann-Whitney U test indicates that male patients with normal or high SMI (> 52.4 cm^2^/m^2^) had a longer ICU length of stay (P < 0.025). Linear regression was calculated to predict ICU length of stay based on SMI. A significant regression equation was found (F (1.96) = 6.823, P = 0.010) (Figure 2), with *R*^2^ of 0.066. SMI was found to significantly predict length of ICU stay (β = 0.258; 95% CI 0.026, 0.189; P = 0.010). The predicted length of stay equals −1.117+0.107 (SMI) days when SMI is measured in cm^2^/m^2^. The ICU length of stay increased by 0.107 days for each cm^2^/m^2^ of SMI in the male population.

A Spearman's rank-order correlation showed no difference in hospitalization time between males with low or normal SMI. Mann-Whitney U test indicates statistically significant differences between low or normal SMI (*P* < 0.05) in more extended hospitalization in male patients with normal SMI. In addition, the linear regression analysis was performed to predict the length of hospital stay based on SMI in male patients; however, no statistically significant regression equation was found (β = −0.031; 95% CI −0.656, 0.480; *P* > 0.05). In the female population, we found no statistically significant influence of SMI on ICU length of stay (β = −0.123; 95% CI −0.741, 0.463; *P* = 0.544) and hospitalization time (β = 0.013; 95% CI 0.075, 0.941; *P* = 0.843).

### Influence of MI on hospitalisation

The Mann-Whitney U test indicates that there were no differences among the groups (MI and no-MI) regarding the ICU length of stay (*P* = 0.161) or hospitalization (*P* = 0.771).

### Influence of SMI and MI on postoperative complications

Postoperative complications of stage 2 or more by Clavien-Dindo classification^19^ were present in 79 patients (57.2%). Infection occurred in 59 patients (42.8%), most commonly as intra-abdominal infection (50.8%), respiratory tract infection (23.7%) and urosepsis (6.8%). 5 (3.6%) patients developed critical illness myopathy. Surgical intervention was needed in 38% of patients with postoperative complications.

There was no statistically significant difference in the frequency of postoperative complications between males (*P* = 0.883) and females (*P* = 0.113) with low or normal SMI. The postoperative infection rate was similar in males (*P* = 0.293) and females (*P* = 0.285) with low or normal SMI. MI did not show significant influence on postoperative complications (*P* = 0.839) and infection rate (*P* = 0.703).

### Influence of SMI and MI on liver graft rejection

Rejection was diagnosed in 22 patients (16.1%), 20 in male and 2 in female patients. We found no statistically significant influence of SMI (males, *P* = 0.875; females, *P* = 0.135) and MI (*P* = 0.449) on liver graft rejection.

### Influence of ME LD and ALBI score on postoperative outcomes

The median MELD score was 14 (7 – 46). A Spearman's rank-order correlation was run to determine the relationship between the MELD score and ICU length of stay (days). There was a weak, positive correlation between the MELD score and ICU length of stay, which was statistically significant (r_s_ = 0.261, *P* < 0.002). There was no significant relationship between the MELD score and complication rate or length of hospitalization (*P* > 0.05).

There is a week, positive correlation between the ALBI score and ICU length of stay and hospitalisation time, which was statistically significant (r_s_ = 0.279, *P* < 0.001; r_s_ = 0.197; *P* = 0.022). There is no correlation between the ALBI score and postoperative complications.

### GLIM score

#### GLIM score using skeletal muscle index as a phenotypic factor

We analyzed differences between patients who meets criteria for positive GLIM score (acute or chronic liver disease as etiological factor and skeletal muscle index as a phenotypic factor) and those with negative GLIM score regarding ICU length of stay, postoperative complications, rate of infections, graft rejections and mortality.

Based on the chi-square independence test, no association was found between the groups in postoperative complications (χ^2^(1) = 0.600; P = 0.438), rate of infections (χ^2^(1) = 0.918; P = 0.338), graft rejection (χ^2^(1) = 1.205; P = 0.272), and mortality (χ^2^(1) = 0.232; P = 0.630). In addition, there were no statistically significant differences in ICU length of stay between groups (χ^2^(14) = 15.125; P = 0.370), based on the Mann-Whitney test.

#### GLIM score using myosteatosis as a phenotypic factor

We analyzed differences between patient who meets criteria for positive GLIM score (acute or chronic liver disease as etiological factor and myosteatosis as a phenotypic factor) and those with negative GLIM score regarding ICU length of stay, postoperative complications, rate of infections, graft rejections and mortality.

Based on the chi-square independence test, there was no association between the groups in terms of postoperative complications (χ^2^(1) = 1.378; P = 0.242), infection rate (χ^2^(1) = 2.921; P = 0.089), graft rejection (χ^2^(1) = 0.873; P = 0.352), and mortality (χ^2^(1) = 0.010; P = 0.922). In addition, there were no statistically significant differences in ICU length of stay between groups (χ^2^(14) = 16.271; P = 0.297), based on the Mann-Whitney test.

### Multiple linear regression analysis

Multiple linear regression was calculated to predict ICU length of stay on SMI and infection. A significant regression equation was found (F (2. 95) = 11.192, P < 0.000), with R^2^ of 0.191. SMI was found to significantly predict length of ICU stay (β = 0.292; 95% CI 0.045, 0.199; P = 0.002). The length of ICU stays increased by 0.122 days per cm^2^/m^2^ SMI in male patients with infection.

### Mortality

In our study group, 5 (3.6%) patients died in the first 90 days after liver transplantation. Two patients died immediately after the procedure due to irreversible haemorrhagic shock with disseminated intravascular thrombosis and abdominal organ ischemia. The cause of death in three patients was sepsis with multiorgan failure in one patient (day 66), and severe postoperative bleeding, intraabdominal infection with liver abscess, and multiorgan failure in two patients (34 and 53 days). One year survival rate was 95%. Due to the small number of patients, statistical analysis was not performed.

## Discussion

Malnutrition with skeletal muscle mass loss is a frequent complication in patients with chronic or end-stage liver disease.^25^ The aetiology of malnutrition is multifactorial.^23^ Insufficient calorie intake (early satiety, loss of appetite, alcohol consumption, diet restriction), metabolic abnormalities, catabolic state of metabolism, and malabsorption are the main contributors to muscle loss.^25,26^ It affects from 30 to 70%^27,28^ of patients with end-stage liver disease and up to 80%^27^ of patients with alcohol-related liver disease. Myosteatosis affects more than half of patients with chronic liver disease.^9^ In our patient population, we used selected parameters for low SMI (men < 52.4 cm^2^/m^2^, women < 38 cm^2^/m^2^)^9,21^ and MI (< 33 HU in patients with a BMI ≥ 25 kg/m^2^ and < 41 HU in those with a BMI < 25).^22,23,24^ These cut-off values were determined specifically for patients on LT waiting list are the most widely used in literature.^22,23,24^ Low SMI was present in 63% of males and 28.9% of females. More than one-third of the patients (32.6%) had fatty infiltration of the skeletal muscle. Patients with alcoholic aetiology had more fat-infiltrated muscles than other aetiologies for end-stage liver disease (P < 0.008). Alterations in the metabolism of fatty acids, eating habits, and alcohol consumption probably result in fatty infiltration of skeletal muscle fibres. However, due to the small numbers of patients (3 patients) with non-alcoholic steatohepatitis (NASH) or non-alcoholic fatty liver disease (NAFLD) in our study group, we cannot properly assess the relationship between the aetiology of underlying liver disease and the degree of MI.

Several studies have shown that sarcopenia and myosteatosis are associated with a higher incidence of postoperative complications, infection rate, duration of hospitalization, and mortality in a wide range of gastrointestinal cancers, including tumours of the hepatopancreatobiliary system.^29,30,31,32^ Lack of a strictly defined patient population, different methods of measurement of muscle mass and function, and the absence of uniformly accepted cut-off points for patients with chronic or end-stage liver disease are the leading causes for wide ranges of sarcopenia incidence reported in the literature.^14,21,33^ Therefore, the selected cut-off points for patients with cancer are used for evaluation, which may affect the quality of results.

Sarcopenia is associated with poorer outcomes in patients with chronic liver disease on the wait-list or after LT.^34,35^ Patients with end-stage liver disease and sarcopenia have shorter survival than non-sarcopenic patients (22 ± 3 *vs*. 95 ± 22-month, P < 0.001).^9,36^ The main cause of death in patients with end-stage liver disease is sepsis.^37^ Incidence of sepsis as a cause of death is even higher in presence of sarcopenia (36% *vs*. 16%, P < 0.001) or myosteatosis (32% *vs*. 19%, P = 0.020).^9^ Lower muscle mass is also associated with a higher incidence of hepatic encephalopathy in patients with end-stage liver disease.^38,39^ On the other hand, a group from USA^40^ showed that sarcopenic patients had a higher tendency for pulmonary complications than the nonsarcopenic group (38% *vs*. 18%, P = 0.100); however, there was no significant difference in morbidity and mortality.

CT imaging is considered as the gold standard for body composition assessment. SMI calculated on the level of third lumbar vertebra is superior since it correlates best with the actual quantity of the muscles in the body.^41^ It is not affected by ascites and is part of preoperative evaluation in patients with hepatocellular carcinoma or the complications of portal hypertension. The value of SMI as a predicting factor for postoperative morbidity and mortality is yet to be determined. However, it has been shown in several reports that it has a negative impact on postoperative outcomes. Lower values of SMI are associated with more significant postoperative mortality, higher infection risk, and postoperative complications, more extended intensive care unit stay, ventilator dependency, and higher waitlist mortality rates in patients with end-stage liver disease.^33,42,43^ SMI as a predicting factor for higher mortality rate in patients with end-stage liver disease was superior to other nutritional assessment indicators, such as BMI, upper arm muscle circumference, and triceps skinfold thickness.^44^ However, some other reports showed contrasting findings. Using SMI for prespecified definitions of sarcopenia had no impact on mortality or delisting from the transplant waitlist between patients with and without sarcopenia.^45^ Length of hospitalization following LT, days of hospitalization during the first year post-LT, survival at one year, or overall survival was not different between sarcopenic and nonsarcopenic patients.^46^ Low SMI alone was not associated with graft and patient survival (P = 0.273 and P = 0.278) after LT.^47^ These conflicting results are probably due to heterogeneity of used specific cut-off points (sex, age, race) and lack of strictly defined parameters representative uniquely for patients with chronic liver disease. We found a negative correlation between BMI and SMI, probably due to high-volume ascites in the patient population This confirms findings that BMI is not suitable for body assessment in patients with end-stage liver disease. In our study, the presence of ascites was associated with significantly lower SMI (P < 0.05) in patients with end-stage liver disease but did not influence MI. There is a weak, positive correlation between SMI and ICU length of stay in male patients, which was statistically significant (rs = 0.226, P < 0.025). We speculate that the main reason is a more pronounced systemic inflammatory reaction to surgical stress in patients with preserved muscle mass. It is known that muscle mass is mandatory for the normal function of the immune system.^48^ Experience from Japan^49^ shows a connection between low SMI and decreased incidence of graft rejection in living-donor liver transplantation. Analysis of our results shows no statistically significant difference in postoperative complications, rate of postoperative infection, and liver graft rejection rate between males and females with low or normal SMI (P > 0.05). BIA is commonly used technique in body composition analysis in every day clinical practice.^50^ Its key parameters are resistance, reactance and phase angle (PA). PA is found to be associated with outcomes in different diseases and has been found to be useful for monitoring fluid changes and response to interventions.^50^ The main limitation is the complexity of the determinants that requires its adjustment to the individual phenotypic diagnosis of each patient. Results can be affected by altered water and electrolyte balance, fluid retention and diuretic therapy.^50^ DXA allows for the quantification of three body compartments (bone mass, fat mass, and bone fat-free mass (or lean mass)) based upon the differential tissue attenuation of X-ray photons.^51^ However, it can be affected by presence of ascites.^52^ Even though DXA can be modified to exclude influence of ascites or tissue oedema the correlation between the lean mass index and SMI was weaker (γ = 0.29, p = 0.035) and falsely high in patients with ascites before liver transplantation.^53^ Main disadvantages of DXA compared to CT is inability to assess muscle mass quality (myosteatosis).^51^

Myosteatosis is a more clearly defined factor, and its influence is more uniformly established in literature.^54^ However, there are many different cut off points (HU < 25 to HU < 39) reported in literature in patients with malignant disease.^55^ Majority of reports uses a Martin cut-off point (< 33 HU in patients with a BMI ≥ 25 kg/m^2^ and < 41 HU in those with a BMI < 25)^56^ for determining presence of myosteatosis. Martin cut-off point is also recently defined threshold parameters for MI in a patient with a chronic liver disease.^22,23,24^ It has been shown that severely ill patients with myosteatosis have a lower survival rate than those without fatty infiltration in muscles.^57^ Myosteatosis negatively impacts the survival of patients with end-stage liver disease (28 ± 5 *vs*. 95 ± 22-month, P < 0.001)^9,36^ and is associated with longer hospitalization and higher morbidity.^43^ Patients with myosteatosis showed a higher mortality rate, most commonly due to respiratory and septic complication.^47^ Myosteatosis had no influence on ICU length of stay (P = 0.161), hospitalisation (P = 0.771), postoperative complications (P = 0.839), infection rate (P = 0.703) and graft rejection (P= 0.449) in our patient population.

The nutritional status on the waitlist for LT as a possible risk factor is overlooked with the MELD score.^58^ Another important limiting factor of the MELD score is using serum creatinine levels for score calculation.^3^ Serum creatinine level may vary significantly and is influenced by chronic kidney disease, ascites, paracentesis, and the influence of gender and liver disease on skeletal muscle mass.^59^ Variation of serum creatinine levels may affect the MELD score and underestimate the severity of liver disease.^58^ Thus, several modifications of the MELD score were developed to incorporate the nutritional parameters. Body composition MELD (BC-MELD)^60^ has a better predictive value for waiting list mortality than MELD score. MELD-sarcopenia score showed a positive predictive value in patients with a lower score (< 15) on the postoperative course; however, it was not useful in patients with MELD above 15.^61,62^ Combining SMI in MELD in multivariate analysis (AUROC= 0.812) is significantly better than MELD alone (AUROC = 0.787) for predicting 5-year mortality (P < 0.001).^44^ We found a weak, positive correlation between the MELD score and ICU length of stay (rs = 0.261, P < 0.002). ALBI score showed promising results in more accurate prediction of liver disease severity and mortality on waitlist compared to the Child-Pugh score; however, its predictive value was inferior to the MELD score.^63^ Patients with pre-transplant ALBI grade 3 liver disease had increased mortality after LT.^64^ We found a weak, positive correlation between the ALBI score and ICU length of stay and hospitalization time, which was statistically significant (r_s_ = 0.279, P < 0.001; r_s_ = 0.197; P = 0.022). We found no correlation between the ALBI score and postoperative complications. The GLIM score^16^ was established as a potential assessment tool for evaluating patient nutritional status in recent years. Our results showed no difference between the patients who met GLIM criteria and those who did not, regarding ICU stay, length of hospitalization, postoperative complications, infection, and mortality.

We believe that nutritional status in a patient with end-stage liver disease is an essential aspect of pre-transplant workup. However, its precise role is not yet determined. LT is a complex surgical procedure influenced by numerous factors. In the early phase of LT, donor characteristics and comorbidities, quality of liver graft retrieval, liver graft quality, cold preservation duration, ischemic damage to the liver graft during static cold storage, recipient medical conditions and comorbidities, surgical procedure, and early postoperative therapy are by our opinion the most essential factors. However, muscle mass status, muscle function and myosteatosis are crucial factors in a period of rehabilitation.^65^

Patient with cachexia or with high-risk (< 18.5 kg/m^2^, Child-Pough C)^66^ to lose muscle mass should be screened and involved in intensive but personalized nutritional support therapy. Nutritional requirements in end stage liver disease are 35 kcal/kg per day in non-obese patients (BMI 30 kg/m^2^) and 1,2g/kg per day intake of proteins.^66^ It is safe to calculate requirements based on the dry weight of a patients.^66^ In presence of hepatic encephalopathy (HE), animal protein should be substituted with vegetable protein origin. Randomized control study^67^ showed that substitution of animal protein with vegetable protein for a period of six months (30–35 kcal/kg/d,1.0–1.5 g/kg/d protein) improved neuropsychiatric performance in patients with minimal HE and decrease their risk of developing overt HE compared to no intervention. It is unreal to expect to gain muscle mass in cirrhotic patients, but muscle mass preservation should be focus of such nutritional interventions. There are several strategies to prevent muscle mass loss in patients with end stage liver disease. First strategy is nutritional supplementations. Patients should have frequent small meals to avoid prolong fasting period (> 6 h).^66,68,69^ Enteral supplements with side branched amino acids should be administered.^66,68,69^ Snacks rich in carbohydrates should be taken as a late-night snack.^66,68,69^ Second, physical activity in a form of resistance and endurance exercise, is probably appropriate and beneficial.^70^ Micronutrition, especially administration of fat-soluble vitamins^71^ and ammonia lowering therapy^72^ is also important. Role of immunonutrition in patient with end stage liver disease is not yet established.^66,73^ Post-transplant screening is advised in all patients after liver transplantation.^66,73^

There are several drawbacks to our study. The two most important are the retrospective nature of data collection and the lack of functional assessment of recipients’ muscles. Another factor that may influence results is the relatively small number of cases that may affect some results or may not produce statistical significance due to the complexity of the treatment and numerous factors that determine its outcome.

In conclusion radiological assessment of a patient's nutritional status at the third lumbar vertebra represents an objective and reproducible method. It should become a standard screening tool in patients with acute or chronic end-stage liver disease. Due to complexity of liver transplant procedure, liver graft and liver recipients’ factors, it is difficult to established impact of a skeletal muscle index and myosteatosis on postoperative outcomes. However, nutritional interventions and physical activity should be part of the clinical pathway in patients with end-stage liver disease waiting for liver transplantation. Prospective randomized controlled studies are not possible due to ethical considerations; hence, standardization and uniformity in definitions, methods, and cutoff points are crucial to producing reliable data in the future.
